# Revisiting Five Years of CASMI Contests with EPA Identification Tools

**DOI:** 10.3390/metabo10060260

**Published:** 2020-06-23

**Authors:** Andrew D. McEachran, Alex Chao, Hussein Al-Ghoul, Charles Lowe, Christopher Grulke, Jon R. Sobus, Antony J. Williams

**Affiliations:** 1 Oak Ridge Institute for Science and Education (ORISE) Participant, 109 T.W. Alexander Drive, Research Triangle Park, NC 27709, USA; chao.alex@epa.gov (A.C.); halghoul88@gmail.com (H.A.-G.); 2 Center for Computational Toxicology and Exposure, Office of Research and Development, U.S. Environmental Protection Agency, 109 T.W. Alexander Drive, Research Triangle Park, NC 27709, USA; lowe.charles@epa.gov (C.L.); grulke.chris@epa.gov (C.G.); sobus.jon@epa.gov (J.R.S.)

**Keywords:** non-targeted analysis, high-resolution mass spectrometry, mass spectral fragmentation prediction, compound database, spectral library

## Abstract

Software applications for high resolution mass spectrometry (HRMS)-based non-targeted analysis (NTA) continue to enhance chemical identification capabilities. Given the variety of available applications, determining the most fit-for-purpose tools and workflows can be difficult. The Critical Assessment of Small Molecule Identification (CASMI) contests were initiated in 2012 to provide a means to evaluate compound identification tools on a standardized set of blinded tandem mass spectrometry (MS/MS) data. Five CASMI contests have resulted in recommendations, publications, and invaluable datasets for practitioners of HRMS-based screening studies. The US Environmental Protection Agency’s (EPA) CompTox Chemicals Dashboard is now recognized as a valuable resource for compound identification in NTA studies. However, this application was too new and immature in functionality to participate in the five previous CASMI contests. In this work, we performed compound identification on all five CASMI contest datasets using Dashboard tools and data in order to critically evaluate Dashboard performance relative to that of other applications. CASMI data was accessed via the CASMI webpage and processed for use in our spectral matching and identification workflow. Relative to applications used by former contest participants, our tools, data, and workflow performed well, placing more challenge compounds in the top five of ranked candidates than did the winners of three contest years and tying in a fourth. In addition, we conducted an in-depth review of the CASMI structure sets and made these reviewed sets available via the Dashboard. Our results suggest that Dashboard data and tools would enhance chemical identification capabilities for practitioners of HRMS-based NTA.

## 1. Introduction

Small molecule identification strategies using high resolution mass spectrometry (HRMS) data are continuously evolving [1,2,3,4,5]. Despite rapid growth in the field, enhancing certainty in structural assignments [6] and establishing cohesive workflows [7] remain persistent challenges. Interconnected data, software, tools, and databases are required to address these challenges, but identifying the best and most fit-for-purpose tool(s) is a daunting task [8,9]. One means by which to address identification challenges, evaluate tools, and share data is through collaborative trials and contests. Over the last decade, trials and contests designed for HRMS-based non-targeted and suspect screening analysis (NTA, SSA) applications have been conducted using environmental samples (water [2], dust [10], etc.), synthetic chemical mixtures [11,12], and MS/MS spectra (e.g., [13,14]). While objectives and goals differ, these challenges and contests have been successful in identifying the leading tools and software and highlighting lingering obstacles for global users.

The Critical Assessment of Small Molecule Identification (CASMI) contest originated in 2012 to provide mass spectrometrists with a means to share and evaluate structure identification tools, software, and workflows on a common, open dataset [13]. Since 2012, four other CASMI contests have been conducted, resulting in multiple special issues, hundreds of citations, and unique data sets upon which researchers can evaluate their own structure identification tools and workflows [14,15,16]. The most commonly used tools in HRMS-based screening experiments (e.g., MetFrag [17], Competitive Fragmentation Modeling for Metabolite Identification (CFM-ID) [18], SIRIUS [19]) have been vetted via the CASMI contests, providing performance benchmarks upon which new analysis tools can be evaluated.

The US EPA’s CompTox Chemicals Dashboard (the “Dashboard”) has recently been established as a valuable resource for the scientific community [20], capable of contributing to confident structural identifications in NTA studies [21]. Preliminary evaluations of the Dashboard have been performed, with evidence indicating that the Dashboard outperforms larger, more popular databases when using data source ranking for identification of unknowns [22]. Additionally, the generation and linking of “MS-Ready” structures [23] has enhanced database searching functionality, and provides access to critical compound metadata. Achieving the highest confidence identifications in NTA studies, however, requires corroborating MS/MS data. Using the open-source CFM-ID MS/MS prediction algorithms, spectra for ~760,000 chemical structures were previously predicted, linked to MS-Ready structures and metadata, and made openly available for the community [24]. Chao et al. evaluated the use of these spectra in a retrospective analysis of the EPA’s Non-targeted Analysis Collaborative Trial (ENTACT) data and showed that combining predicted MS/MS spectra with curated empirical spectra improved the successful identification rate [25].

The Dashboard was initially released in April 2016, and its first application to HRMS research was published in 2017 [22]. The Dashboard was too new and immature in functionality to participate in any of the five previous CASMI contests. However, with the implementation of relevant schemes for candidate ranking, improved search functionality, and predicted MS/MS data, evaluation of the Dashboard for HRMS-based screening purposes is now possible. We have therefore computed results for all five CASMI contest datasets using only Dashboard tools for the analysis. Here, we present the workflow, methods, and results utilizing Dashboard tools for all five CASMI contests. This work highlights the strengths and weaknesses of the Dashboard for HRMS-based screening studies and provides a commentary on needs for data compilation and curation within the NTA research community.

## 2. Results and Discussion

### 2.1. Dataset Assembly

An investigation was performed on each of the individual CASMI datasets to identify whether the chemicals were present in DSSTox, the database underlying the Dashboard (see Methods for details). As our identification workflow relies on database presence, the presence or absence of a chemical in the database is clearly highly influential in terms of overall performance. Results from the dataset assembly analysis from each CASMI year dataset are presented in Table 1 and described in detail by dataset year below (complete datasets are provided in Supplemental File S1 and will be available as lists on the Dashboard in a future release).

#### 2.1.1. CASMI 2012

A total of 14 challenge chemicals were present in the 2012 dataset associated with LC-MS analyses (http://casmi-contest.org/solutions.shtml) [26]. Only one chemical in the dataset had two associated stereoforms in the Dashboard (reticuline). Two of the chemicals had different InChIKeys based on representations of the C = N bond with the crossed bond being the preferred form. Five structures were registered in the database to complete the assembly of this dataset (Table 1).

#### 2.1.2. CASMI 2013

A total of 16 challenge chemicals (http://casmi-contest.org/2013/solutions-cat1-2.shtml), plus three specific tautomeric forms for Challenge 11 (demethoxycurcumin), were present in the 2013 dataset. Based on a first block InChIKey search, three chemicals returned multiple hits (all due to stereoform differences) and nine chemicals in the dataset returned InChIKeys that did not match the CASMI InChIKey. Two had different InChIKeys based on the representation of the C = N bond with the crossed bond being the preferred form. Close inspection showed, for example, that in certain cases CASMI InChIKeys did not match the associated chemical name (e.g., Challenge 7, cinnamtannin A3 had no stereochemistry encoded in the input InChIKey while the structure contains multiple stereocenters, as shown in https://comptox.epa.gov/dashboard/DTXSID70904147). Eight structures were registered in the database to complete this dataset (Table 1).

#### 2.1.3. CASMI 2014

A total of 42 challenge chemicals in the 2014 dataset were associated with LC-MS analyses (http://casmi-contest.org/2013/solutions-cat1-2.shtml). Ten of the input InChIKeys returned multiple hits following a Dashboard search (seven with two hits and three with three hits each). Furthermore, a total of 21 mismatches were observed between the input InChIKeys and the InChIKey returned from the Dashboard search. For Challenge 29, californidine, the CASMI InChIKey represented the chloride salt (https://www.ebi.ac.uk/chembl/compound_report_card/CHEMBL538357/) rather than the parent cation (charged as a result of the tetravalent bridgehead nitrogen). Thirteen structures were registered in the database to complete this dataset (Table 1).

#### 2.1.4. CASMI 2016

Two datasets were associated with the CASMI 2016 contest: 312 chemicals were in the training dataset and 208 were in the challenge dataset (http://casmi-contest.org/2016/challenges-cat2+3.shtml). For the training dataset, 48 of the input InChIKeys returned multiple hits from the Dashboard (between two and five hits each) and a total of 86 mismatches were observed between the input InChIKey and the result set returned from the Dashboard. For the challenge set, 31 of the input InChIKeys returned multiple hits (between two and four hits each) and a total of 51 mismatches were observed between the input InChIKey and the result set. No structures needed to be added to the database in order to complete these datasets (i.e., every input InChIKey returned a hit).

**Figure 1:**
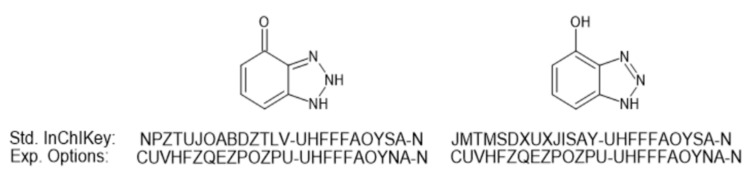
The standard InChIKeys for the tautomeric keto and enol forms of 4-hydroxybenzotriazole. Also indicated are the experimental InChIKeys generated using ACD/ChemSketch to account for keto-enol tautomerism.

Close analysis of these specific datasets provides insight into some of the challenges associated with tautomeric forms and database searching. As an example, the structure associated with 4-hydroxybenzotriazole (Training-094, https://pubchem.ncbi.nlm.nih.gov/compound/135399369) is represented in the associated spreadsheet with the SMILES for 1,2-dihydro-4H-benzotriazol-4-one (https://pubchem.ncbi.nlm.nih.gov/substance/363592962). There are two submitters to PubChem for the keto form based on a search using the provided InChIKey (https://pubchem.ncbi.nlm.nih.gov/#query=NPZTUJOABDZTLV-UHFFFAOYSA-N): MassBank of North America (MoNA) and the NORMAN Network. However, the tautomeric form of this chemical does not have the same Standard InChIKey for the individual keto and enol forms and is thus not collapsed via standard InChI tautomerization. It should, however, be noted that there are experimental options for InChI generation for 1,5-tautomerization and keto-enol tautomerism (available within ACD/ChemSketch and discussed in https://www.inchi-trust.org/technical-faq-2/) that do provide equivalent InChIKeys as shown in Figure 1. However, as yet these do not appear to be used in any public databases, as a Google Search provided no hits for the collapsed InChIKey. It is acknowledged that not all databases and their associated InChIKeys are necessarily indexed by Google. Mismatching due to tautomers not collapsing based on InChIKey also occurred for clozapine (Training-143, Supplemental File S2, Figure S1).

Issues regarding mapping between tautomers are common in public databases and require careful curation to manage. Examples of confusion resulting from the mixing of data for the two individual tautomeric forms can be demonstrated by reviewing the results set in EBI’s UniChem for a search of the InChIKey pairs for clozapine (https://www.ebi.ac.uk/unichem/frontpage/results?queryText=ZUXABONWMNSFBN-UHFFFAOYSA-N%0D%0AQZUDBNBUXVUHMW-UHFFFAOYSA-N&kind=InChIKey&sources=&incl=exclude), the variations of ChemSpider names for 4-hydroxytriazole (http://www.chemspider.com/2040044), and the differences in the identifiers versus the structure representation for 4-hydroxytriazole in a NORMAN Network Fact Sheet (see Supplemental File S2, Figure S2).

#### 2.1.5. CASMI 2017

The CASMI 2017 challenge was focused on Natural Products. In the 2017 solutions file downloaded from the CASMI site there were a total of 243 challenge chemicals in the dataset associated with LC-MS analysis (http://casmi-contest.org/2017/solutions.shtml). Inspection of the solutions set indicates that many of the structures that would be expected to have explicit stereochemical forms have no stereo as defined by block 2 of the provided InChIKeys. Since the curation effort for the assembly of chemical structures underlying the Dashboard (the DSSTox database) attempts to include stereochemistry to match the relevant identifiers (CASRN, name, etc.) it is to be expected that matches using a first block search would retrieve full stereo representations. An InChIKey first block search for the set of challenge InChIKeys retrieved over a hundred structures with explicit stereochemistry indicating that many, but not all, of the challenge chemicals were submitted without stereochemistry.

Thirty of the input InChIKeys returned multiple hits (between two and three hits each) based on a first block search and a total of 84 mismatches were observed between the input InChIKey and the result set returned from a Dashboard search. In the curated spreadsheet of 243 challenge chemicals, 13 of the input InChIKeys had no resulting hits in the Dashboard based on a first block search. It is noted that *none* of these natural products had associated stereochemistry despite several having complex carbohydrate substructures. An Internet search indicated that the vast majority of these compounds could be found only in the online CASMI dataset itself and on PubChem associated with CASMI (i.e., the CASMI dataset, and places where indexed, appear to be the primary online source of the InChIKeys). A search for the full InChIKeys in UniChem showed that 8 of the 13 were present in a narrow number of sources (see Supplementary Table: UNICHEM_CASMI2017) and are all known to sample from each other’s data resulting in self-referencing: PubChem [27], ZINC [28], MCule [29], eMolecules (https://www.emolecules.com/info/plus/download-database), and MolPort (https://www.molport.com/shop/database-download). All 13 chemicals were retrieved in the MassBank of North America dataset associated with an original source of the Global Natural Product Social Molecular Networking Library (GNPS). A review of chemical representations on GNPS (https://gnps.ucsd.edu/) indicates that “incomplete” chemical representations, lacking explicit stereochemistry, are common, potentially because the stereo details are not known or simply because it was not included in the structure drawing during registration. One-hundred-and-seventy-three structures were registered in our DSSTox database matching either the chemical name or matching skeletal forms (i.e., matching the first block of the InChIKey), and including associated trivial names and registry numbers where available. This enhanced the dataset for the purpose of this study, but it remains incomplete as we did not include chemicals from GNPS where no representations of the chemicals that were expected to have multiple stereochemical centers were found. Including structures such as these would detract from our ongoing effort to improve the quality of data in our database.

#### 2.1.6. Dataset Assembly Summary and Ongoing Work

**Figure 2:**
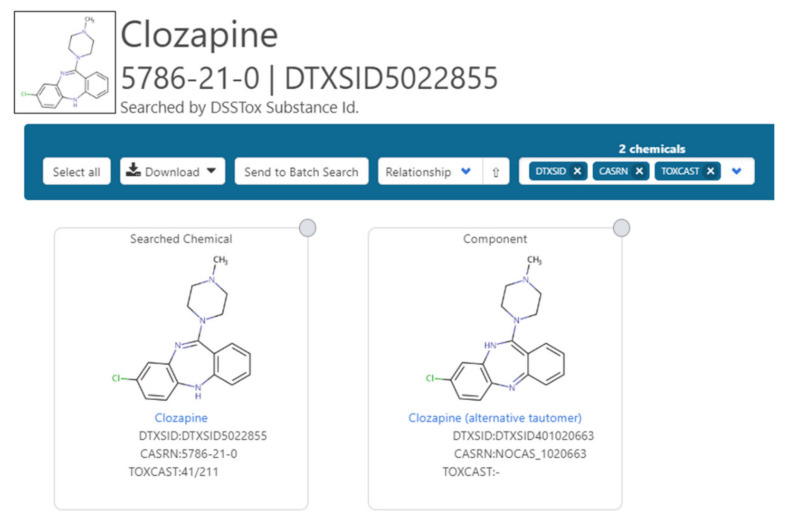
The two tautomers of clozapine registered in the database. This is possible because the data do not collapse based on standard InChIKeys. They are mapped against each other as related substances.

Ongoing curation efforts coordinated between the NORMAN Network and the EPA around chemical mappings continue to focus on inconsistencies with the intention of ultimately elevating integration across all platforms: for example, where MassBank Europe has the enol rather than the keto representation of a chemical (https://massbank.eu/MassBank/Result.jsp?inchikey=JMTMSDXUXJISAY-UHFFFAOYSA-N). This includes the registration and mapping of alternative tautomers that do not collapse by standard InChIKey (as illustrated in Figure 2 for clozapine). Each of the individual tautomers is mapped to the alternative tautomer so that they are viewable as related substances. Registration of both tautomers ensures retrieval based on the individual InChIKeys as search inputs. The enumeration and registration of all potential tautomers that do not collapse based on InChIKey enumeration for all chemicals in the Dashboard would lead to a combinatorial explosion and this example is included for completeness only to demonstrate feasibility. It is hoped that tautomeric collapse through Standard InChI will continue to improve with time, and efforts are underway as evidenced by the keto-enol and 1,5-tautomerism experimental settings that are available for InChI at present. Alternatively, such challenges may potentially be addressed with additional pre-processing standardization efforts prior to InChI generation. The development of a new MS-Ready standardization library is presently underway and testing shows that in both cases these tautomers do collapse and generate the same InChIKey.

Several issues were observed during dataset assembly. Certain issues focused on structural correctness in terms of the appropriate structural representations, while others were more hypothetical: i.e., it is not possible to know specifically what stereoform of a chemical was represented by a mass spectrum alone and in some cases this had to be inferred from relevant metadata. While several issues were identified with the datasets, this is not meant to be a criticism of the assembled data. The CASMI contests have provided some of the most useful datasets, with associated publications (e.g., [13,16,30,31,32,33]) made available to the community to test and validate identification workflows. The scope of this multi-year project has been expansive in nature, attempting to test existing capabilities and approaches for structure identification across a number of scenarios. The assembly of highly-curated datasets remains an extremely challenging undertaking and, still, cheminformatics approaches remain limited especially in terms of structure standardization and the full applicability of InChIs and their associated keys to represent tautomeric forms. The accurate representation of chemical structures in many online databases is both an issue of the work required to ensure accurate representation, the provenance of the source(s) of the information used as a reference to provide this representation, and the common ambiguity of the names used to represent a particular chemical. The work required just to assemble the correct structures associated with the set of spectra associated with the CASMI contests would require significant time and ultimately a consensus agreement between the organizers. Our efforts here have been to attempt this work post factum without the engagement of the original organizers to assist in identifying what the chemicals were supposed to be. We hope that the curation output from this work may provide a useful dataset for reference to the CASMI work performed to date.

### 2.2. Individual CASMI Contest Year Results

#### 2.2.1. CASMI 2012

**Figure 3:**
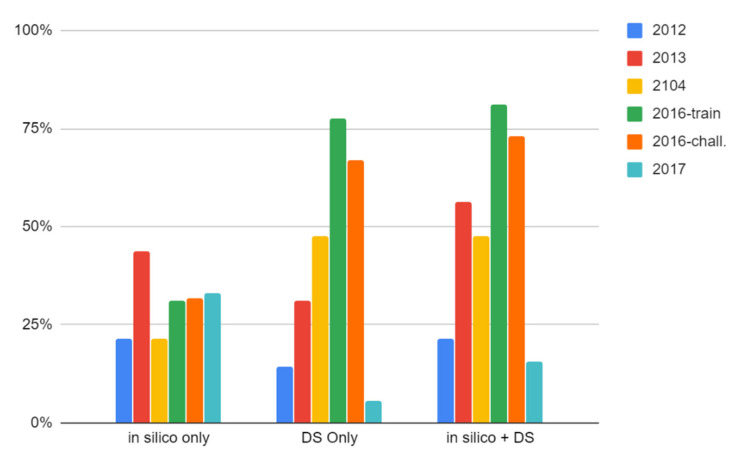
Percentage of challenge compounds from each CASMI dataset ranked in the top (number 1) position by in silico MS/MS match only, Data Source Count (DS) only, and the combined score of in silico MS/MS data with Data Source Counts.

Of the 14 structures in the CASMI 2012 dataset, five were ranked in the top five of all candidates when using only the CFM-ID MS/MS match score and nine were ranked in the top five when combined with Data Source counts (64%, Figure 3). Only 3 out of 14 (21%) were ranked in the top position using the combined scoring metric. Interestingly, 7 out of 14 were ranked in the top five when using Data Source counts alone and all 14 structures were found in the top 10 candidates when using the combined score. The winning entry for CASMI 2012 placed 36% of the challenge structures in the top five and 21% in the top position, matching the number achieved in this work using CFM-ID MS/MS matching alone and falling short of our results when we combined the MS/MS match score with data source counts (Table 2, complete results can be found in the Supplemental Material, Supplemental File S1).

#### 2.2.2. CASMI 2013

Thirteen of the 16 unique structures in the 2013 dataset were ranked in the top five of all candidates using only the MS/MS match score and seven were ranked number one. One compound in this dataset did not have predicted MS/MS data at the time of publication, preventing it from being scored and ranked (Challenge 7, cinnamtannin A3). Adding metadata to ranking only increased the challenge compounds resulting in the top five by one (14 out of 16, Figure 3 and Supplemental File S1). Our results matched the winning entry for CASMI 2013 when considering challenge compounds ranked in the top five but fell short of the winning entry’s number in the top position (12 out of 16, 75%).

#### 2.2.3. CASMI 2014

Twenty-four of the 42 compounds in the 2014 dataset were ranked in the top five of candidates using only MS/MS match scores, and 32 were ranked in the top five when data source counts were added to the scoring and ranking (see Supplemental File S1). One compound in this dataset did not have predicted MS/MS data at the time of publication, preventing it from being scored and ranked (Challenge 31, peonidin). A substantial increase in the number of top-ranked challenge compounds was observed when considering data source counts: only nine were ranked in the top position when using CFM-ID alone, while 20 (48%, see Figure 3) were in the top position when adding data source counts. The winning entry for CASMI 2014 placed 30 in the top five (compared with our 32) and 24 of these were in the top position, thereby outperforming our top-ranking results.

#### 2.2.4. CASMI 2016

Of the 312 compounds in the CASMI 2016 training set, 198 were ranked in the top five using MS/MS match scores alone and 299 were ranked in the top five when additionally considering data source counts. Two-hundred fifty-three (81%, Figure 3) were ranked in the top position using the combined scoring metric. Interestingly, the same number were ranked in the top five when using data sources alone (see Supplemental File S1). This dataset did not have defined winners because the data were intended for use as a training set. The highest performing combination of the work reported in Blazenovic et al. [34] placed 304 in the top five.

One-hundred thirty-eight of the 208 compounds in the 2016 challenge set were ranked in the top five using CFM-ID alone and 196 were ranked in the top five when additionally considering data source counts. One-hundred fifty-two (73%, Figure 3) were ranked in the top position using the combined scoring metric (see Supplemental File S1). Interestingly, 193 were ranked in the top five when using data source counts alone. The winning entry for the 2016 challenge set placed 169 in the top five and 146 in the top position (Table 2), but re-analysis by Blazenovic et al. [34] placed 194 in the top five using their top-performing workflow combination (described in detail below).

#### 2.2.5. CASMI 2017

Finally, 144 of the 243 compounds in the 2017 set were ranked in the top five using CFM-ID match scores alone and 130 were ranked in the top five when combining MS/MS data with data source counts (see Figure 3 and Supplemental File S1). This is the only dataset where the addition of metadata decreased performance. The winning entry from this challenge year placed 180 compounds in the top five and 91 in the top position, using all available data (Table 2). Before database curation and registration, only 54 compounds were in DSSTox and when the identification workflow was conducted on that limited data set, only 38 were ranked in the top five. The 2017 dataset remains only 93% complete in DSSTox as we chose to not register chemicals without any form of explicit stereochemistry where the only source of the chemical appeared to be GNPS and public databases containing the CASMI dataset (see Methods). Additionally, due to the nature of our MS/MS prediction workflow, 52 of the recently added structures did not have predicted MS/MS data at the time of publication, further impacting results. The substantial addition of compounds also explains why simple data source counts did not really increase performance; if the compounds only have one data source then it is likely that other candidates returned from a monoisotopic mass search had a greater number of data sources. The influence of PubChem Data source counts on the final rank of this set was investigated since many compounds in this dataset in DSSTox had only one data source. Adding a normalized score of PubChem counts to the CFM-ID match score also resulted in worse performance. The PubChem reference count is compiled based on presence in underlying datasets. These results suggest that metadata rankings relying on abundant dataset presence are not beneficial when the known compounds are relatively obscure, even when using the much larger PubChem.

#### 2.2.6. Summary

In total, when combining MS/MS data and data source counts, we placed more challenge compounds in the top five of ranked candidates than did the winners of three contest years (with an additional tie for a fourth contest year). We further placed more challenge compounds in the top rank position than did the winner of one contest year (with an additional tie for a second contest year). Despite this performance, without database curation and registration efforts our results would have fallen short for several of the contest years. Database curation and registration had a substantial impact on the 2012, 2013, 2014, and 2017 datasets. Thirty-six percent of the 2012 dataset, 50% of the 2013 dataset, 31% of the 2014 dataset, and 71% of the 2017 dataset were not present in DSSTox prior to registration efforts. As evidenced by the nearly three-fold increase in challenge compounds ranked in the top five of the 2017 dataset before and after database registration, the 2017 dataset represents the most extreme case of database boosting. Database boosting is critical for correct compound identification in many workflows [34], most especially ours. Database presence will therefore remain a biasing factor in NTA workflows for the foreseeable future. It should be noted that the identification tools used by contest participants may have improved since their use in the CASMI contests, especially the earliest years. The CASMI contests and data sets were ideal for evaluation of the Dashboard tools and comparing performance to the results of each contest year was a complementary means to place performance in context.

An identification in the top ranked position is clearly desired, but when handling true unknowns and multiple pieces of supporting information, it is critical to consider where and when results fall outside of the top results. Approximately 9% (75) of compounds in all challenge sets combined were present outside of the top 20 ranked candidates when using the combined MS/MS and data source score (~6% when only considering the compounds present in DSSTox). Sixty-five of the 75 compounds ranked outside the top 20 were from the 2017 set; when removing 2017 only ~2% were outside the top 20. When compounds are not in the top rank position using the workflow described here, additional data must be leveraged to result in a successful identification. Prior analyses suggest that incorporating method-specific information such as chromatographic retention time [35] and structure–method compatibility increase the specific weight of evidence towards correct identifications.

### 2.3. Comparison with CFM-ID Contestants and Post-Contest Evaluations

Throughout the years of CASMI contests, several participants have used CFM-ID for candidate identification. Comparing the results reported by participants using CFM-ID alone versus our MS/MS match score alone indicates that we outperform most of those implementations of CFM-ID. In the CASMI 2013 contest, participants identified two compounds as the top-ranked candidate and placed 10 in the top 10, while we placed 7 and 13, respectively, using the MS/MS match score alone. In the 2014 contest, participants using CFM-ID plus metadata identified 24 compounds in the top-ranked position and 33 compounds in the top 10, while we placed 9 and 28, respectively using the MS/MS match score alone. Finally, in the 2016 contest, participants using CFM-ID alone placed 39 and 123 in the top 1 and 10, respectively while we placed 152 and 201, respectively. We acknowledge that the assembly of the prediction database is not the same as using an in silico tool alone; however this assembly provides unique benefits of inherently restricting candidate chemicals to a curated set instead of relying on the larger and, in our opinion, more diluted PubChem or ChemSpider. Even without adding data source ranking, the assembly of the prediction database alone is advantageous for the identification of unknowns.

The work conducted by Blazenovic, et al. [34] provided another valuable point of reference for further analysis of the CASMI 2016 contest data and results. In their work, the researchers conducted a re-analysis and identification of the CASMI 2016 compounds using four separate in silico tools and added permutations of the models by combining the tools with “database boosting” and, critically, a consensus model of the four in silico tools. For the training set, the best performing model was the combination of “MetFrag + CFM-ID + DB + MS/MS Voting/consensus” and resulted in 290 compounds in the number one ranked position with 305 (out of 312) in the top 10 [34]. Using CFM-ID alone resulted in 48 and 170 in the top position and top 10, respectively while CFM-ID with database boosting resulted in 236 and 295, respectively. For the test set, the best-performing model in Blazenovic et al. was “CFM-ID + ID_sorted + DB + MS/MS Voting/consensus” and resulted in 181 in the top ranked position and 201 (out of 208) in the top 10. Using CFM-ID alone on the test set resulted in 29 and 104 in the top position and top 10, respectively, while adding database boosting to CFM-ID resulted in 151 and 191, respectively [34]. The results of our combined ranking workflow resulted in 152 in the number one ranked position and 201 in the top 10 for the challenge set, and 253 in the number one position and 306 in the top 10 for the training set. For both sets, the number of the correctly identified compounds in the top 10 was similar between our combined ranking workflow and the best-performing model reported by Blazenovic et al. However, the number of correct top hits was markedly better in the work by Blazenovic et al. CFM-ID alone performed rather poorly in the work by Blazenovic et al., only ranking ~15% of the compounds in the top rank position, while the MS/MS match score alone in this work placed double the number of compounds in the number one ranked position.

These observations show that the structure of the predicted MS/MS database within the context of the Chemicals Dashboard substantially outperforms running the same in silico MS/MS model with other varied inputs. CFM-ID relies on chemical structure candidate sets for MS/MS prediction and identification workflows. Using the carefully curated and constructed data in DSSTox as the underpinning for candidate sets outperformed sets generated from other sources during the CASMI contests. Further, the simple addition of Data Source counts to the single MS/MS match score from CFM-ID places the same number of compounds in the top 10 as a highly optimized rank model. However, to achieve the highest confidence in top ranked hits, use of more than one in silico model appears to be optimal; i.e., consensus modeling appears to be the best approach.

### 2.4. Spectral Match Interrogation

Scoring relative to an entire set of candidate structures returned from a database query can provide substantial evidence for compound identification. Yet, this method remains biased based on the database from which the candidates were retrieved. Examination of the raw MS/MS match scores provides a means to add additional confidence and awareness during the identification process.

Chao et al. [25] established cutoff values for predicted MS/MS match score results based on a quotient defined as the raw MS/MS match score relative to the maximum match score returned per precursor mass query. Using a global true positive rate (TPR) of 0.90 on a calculated receiver operating characteristic (ROC) curve, Chao et al. defined a quotient cutoff value of 0.13 for guidance towards making informed decisions out of a results set. This quotient cutoff value does not address raw match score values as indicators of an identification outside of the context of database presence; the quotient only places scores in context to other scores returned from the database.

Consider first the quotient values reported in this study. Ninety-four percent of all knowns (true positives) had quotient values greater than or equal to 0.13, closely matching the results observed by Chao et al. and their recommended cutoff [25]. However, summary statistics reveals that both raw and quotient values are study-dependent. Considering results for all identified compounds, the median and mean raw MS/MS match score reported by Chao et al. were 0.58 and 0.33, respectively while the mean quotient value was 0.69. In our work the median and mean raw MS/MS match score of all challenge compounds were 0.65 and 0.80, respectively and the mean quotient value was 0.87.

**Figure 4:**
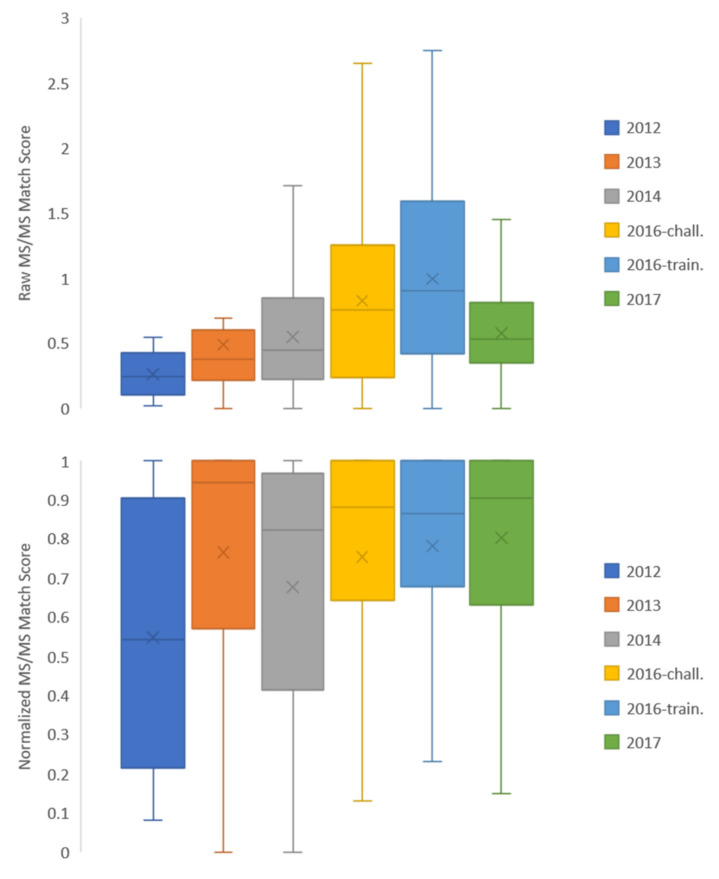
(Top) Raw MS/MS match scores from all compounds in each CASMI year dataset and (Bottom) match scores normalized to the highest score within each candidate set by contest year (the “quotient” term as defined by Chao et al.). Raw scores range from 0 to 3.0 and normalized scores range from 0 to 1.0. Within each contest year, box edges define the 25th and 75th percentile of scores, while the interior line defines the median. “x” markings indicate the mean.

Figure 4 shows raw MS/MS match scores and quotient values separated based on CASMI contest year. Distributions of raw MS/MS match scores show variability from year to year, potentially due to empirical data quality, variability in instrumental conditions, and/or dataset-dependent chemical space weaknesses of the CFM-ID prediction algorithm used to generate spectra. Certain years had quite low match score medians: the 2012–2014 dataset years had a median raw MS/MS match score value less than 0.5 out of a total of 3.0 summed across three collision energies. Despite these low match scores, correct identifications were made at a high rate due to the placement of the correct compound relative to the rest of the candidate set. Normalizing the data by the values returned in each candidate set, the distributions of scores as quotient values were more similar year to year (Figure 4, bottom). This indicates that the true positive value is routinely near the top of the candidate set returned despite a potentially low raw MS/MS match score value: in all years but 2012, half of the compounds had match scores that were at least 80% of the match score of the top-ranking compound (Figure 4, bottom). This discrepancy highlights a challenge for implementation: while the structure of our prediction database enabled a high identification rate by placing the true positive compound near the top of candidate sets across nearly all dataset years, the low raw MS/MS match scores raise a flag for putative identification outside of the context of this database. The ideal situation would be that a compound is more likely to be the “known” than the rest of the candidates from the database (i.e., high quotient value) and that its fragmentation pattern matches well to the predicted data regardless of candidate presence (i.e., high MS/MS match score value). Low raw MS/MS match scores alone are not suggestive of a negative identification; however, the raw value should be flagged when unrealistically low and further interrogated before making an identification. Therefore, it is recommended to carry both the quotient and raw match score values throughout an identification workflow as justification for a positive identification.

## 3. Materials and Methods

The structure of the CASMI contest has been described many times (e.g., see [13,14,15]) and remained consistent despite changes in contest size and chemical space. At the most basic level, individual year contest organizers challenged participants to identify chemical structures using provided un-annotated empirically collected MS/MS spectra through manual and automatic workflows. A total of five contests have been conducted, with the most recent taking place in 2017. All five contests have been evaluated in the current work using our tools and workflows as described earlier. The training set for CASMI 2016 was not used for model optimization as originally intended but was repurposed instead as another evaluation set. By doing so, the total number of trial datasets was increased to six: CASMI 2012, CASMI 2013, CASMI 2014, CASMI 2016-training, CASMI 2016-challenge, and CASMI 2017.

### 3.1. EPA Tools for Structure Identification

The CompTox Chemicals Dashboard and underlying DSSTox Database provided the basis for the structure identification tools used in this analysis. The historical cheminformatics organization of the DSSTox database has provided a unique method for defining chemicals of importance based upon considerations for general commerce and public health [20]. The “Data Source Count” metric provided by the Dashboard represents the number of times a dataset in the underlying DSSTox database contains a particular chemical and has proven a valuable means to prioritize candidate chemicals from HRMS-based screening studies [22]. The database organization also enables access to and linkage between other large databases and screening lists such as PubChem (https://pubchem.ncbi.nlm.nih.gov/), ChemSpider (http://www.chemspider.com/), and the NORMAN Network Suspect lists (https://www.norman-network.com/?q=suspect-list-exchange) [36]. These were investigated as additional scoring metrics (see below).

In order to provide additional confidence in compound identifications, MS/MS spectra were predicted for all MS-Ready structures in the DSSTox database using the open-source CFM-ID models [18,37,38]. The generation, storage, and accessibility of these spectra are described elsewhere [24]. The combination of the Dashboard tools and CFM-ID predicted MS/MS spectra has only recently been described [25], and this work represents an additional investigation into the utility of the combination.

### 3.2. Compilation of CASMI Spectral Files

Spectra and associated data were accessed via the CASMI Contest webpage (http://casmi-contest.org/) for all contest years except 2012, which was accessed via the associated SourceForge page (https://sourceforge.net/p/casmi/web/HEAD/tree/web/challenges/). Available data varied by contest year, but always included individual text files containing MS/MS spectra as *m/z*-intensity pairs for each challenge compound. Spectra were acquired using different instrument conditions depending on the contest year (see associated publications and webpages for more details). For certain contest years (i.e., 2016 and 2017), additional metadata (e.g., precursor *m/z*, retention time) were provided in downloadable .csv files and in other years separate text files containing MS data for each compound were available (e.g., 2014). In order to query the challenge compound MS/MS data against our database of predicted MS/MS data, individual text files were processed and amended for compatibility with in-house spectral matching code developed in Python (http://sourceforge.net/projects/cfm-id). This required extraction of precursor masses from provided data and insertion into the MS/MS text files through additional data manipulation. An example processed file is provided in Supplemental File S3. Processed files were stored by contest year to enable organized database lookups.

### 3.3. Structure Identification, Scoring, and Ranking

Once spectral data were assembled in the correct file format, all data were processed in the same manner. Peak matching and scoring were conducted in custom Python code. A single text file containing challenge MS/MS data was parsed such that the precursor *m/z* could be searched against all MS-Ready monoisotopic masses within the defined error range in the predicted MS/MS database (+/− 10 ppm). For code and query compatibility, precursor ion species were assumed to be [M + H] or [M − H] based on the provided data. The resulting candidate set was returned along with all corresponding predicted *m/z* and intensity pairs. MS/MS data from the input text file were parsed and all individual peaks matched and scored against the stored, predicted data using the cosine dot product metric [39]. The MS/MS peak match threshold used was 0.02 Da. Resulting match scores for all candidate structures were based on three collision energies (10, 20, and 40 eV). Due to the variety of collision energies and instrument types used within and across CASMI contests, match scores from all three collision energies were summed to allow for the greatest coverage of identifications. This results in a raw combined MS/MS match score of up to 3.0 for each candidate compound.

Metadata ranks were used as additional scoring terms for compound identification. DSSTox Substance Identifiers (DTXSIDs) for all candidates returned from each precursor match query were searched via the Dashboard Batch Search to retrieve metadata. Metadata downloaded included DSSTox Data Source Counts, the PubMed Reference Count, the number of PubChem Data Sources, and the presence in the NORMAN SusDat list. Metadata values were linked to all candidate structures returned from the MS/MS match results and normalized from 0 to 1 within each result set. Preliminary investigations revealed that combining multiple counts for ranking did not outperform the use of Data Source Counts alone (see Supplemental File S2, Figure S3). Data Source Counts were thus the sole piece of metadata used in the ranking as described below. Chemicals were ultimately ranked by several different criteria to evaluate the performance of EPA tools for compound identification. Specifically, the ranks of the challenge compounds were organized by Data Source Counts alone, CFM-ID match score alone, and combined CFM-ID match score with Data Source Counts. The combined score was a summation of the normalized Data Source Count and normalized MS/MS match score within each set of candidates. Summary data available from the CASMI Contest webpages were tabulated to determine where the known challenge compounds ranked within our sorted candidate sets (see below for assembly and review of datasets).

### 3.4. Assembly and Review of CASMI Input Data Sets

An investigation was performed on each of the individual CASMI datasets to identify whether the chemicals were present in the Dashboard. As our identification workflow relies on database presence, the presence or absence of a chemical in the database is highly influential in terms of the overall performance. During the review process, an effort was made to assess the accuracy of the associated structures via mapping between the InChIKeys and chemical names, including explicit stereochemistry associated with a specific chemical. While mass spectrometry may be unable to distinguish between various stereoforms of a chemical, it was deemed appropriate to attempt to qualify the CASMI data relative to the data in the Dashboard. When it was determined that chemicals from a CASMI dataset were not present in the database, new chemicals were registered to complete the dataset. This provides a more complete means to evaluate performance and provides clean datasets for the community via the Dashboard. As will be outlined below, many of the chemicals that did match those in the database, based on matching the InChIKey first block, did not match the second block, generally as a result of either incomplete or different stereochemistry. In order to register new chemicals for the purpose of identification, an attempt was made to source an appropriate chemical structure matching the chemical name as the primary identifier. In some cases, especially for natural products (i.e., CASMI 2017), the chemical name was not stereospecific (i.e., no (+)/(−)- or *R*/*S* stereo prefixes). Registration of new chemicals included, where appropriate, multiple stereoforms for a particular chemical based on the name and searching of the InChIKey first block. Several databases were used to source and cross-validate the data. They included ChemSpider (http://www.chemspider.com/), ChEBI (https://www.ebi.ac.uk/chebi/), KnapSack (http://www.knapsackfamily.com/KNApSAcK/), PubChem (https://pubchem.ncbi.nlm.nih.gov/), Wikipedia (https://www.wikipedia.org/), and UniChem (https://www.ebi.ac.uk/unichem/).

Based on previous experiences with data quality and curation issues, specifically around representation of complete and complex stereochemistry, batch searches of the Dashboard (https://comptox.epa.gov/dashboard/dsstoxdb/batch_search) were conducted using the InChIKey skeleton (first block of the InChIKey) of the provided InChIKeys for each of the CASMI datasets. It should be noted that in some cases the CASMI datasets were heterogeneous, containing both standard and non-standard InChIKeys so, where necessary, input InChIKeys were edited from a non-standard to standard form in order to ensure that searches against the dashboard and other public resources (e.g., UniChem) gave the best chance of matched retrieval. The intention of this search was to determine whether one or more chemicals with a specific molecular skeleton was registered in the database. Isotopically labeled chemical forms (e.g., C13 or deuterium labeled) and charged forms retrieved from the first block InChIKey query were excluded from the mappings as they would differ in mass/formula from the input string. Resulting datasets are provided as Supplementary Information and were discussed in detail earlier. The Supplementary Files are enhanced forms of the original files downloaded from the CASMI site. They include new footnotes, color-coded aggregation of chemicals based on the same InChIKey first block, an additional column indicating mismatches between the CASMI input InChIKeys and retrieved hits, a column indicating the associated number of data sources based on an InChIKey lookup against the EBI UniChem service (https://www.ebi.ac.uk/unichem/), and an indication of whether the chemicals are in the latest public release of the Dashboard (as of January 2020).

## 4. Summary and Conclusions

NTA practitioners often rely on multiple tools, databases, and software applications to arrive at a fit-for-purpose identification solution. Using the CASMI Contests, the CompTox Chemicals Dashboard (and underlying data) was evaluated as an identification tool and compared against a variety of tools and workflows dating back to 2012. The datasets themselves provide a valuable resource to the community and were carefully reviewed when mapping to the Dashboard to re-curate as needed for community benefit. Overall, the Dashboard performed extremely well, outperforming participants from certain contest years and coming close in the rest. The greatest boost provided by the Dashboard and supporting data comes from the underlying organization as it relates to data source ranking and structure of the predicted MS/MS data in a confined space. These results suggest that NTA practitioners would benefit from incorporating the Dashboard into workflows where appropriate.

## Figures and Tables

**Table 1:** Number of structures in each Critical Assessment of Small Molecule Identification (CASMI) contest year and the number of those present in DSSTox before and after database registration.

Contest Year	Total Number of Challenge Structures	Total Number of Structures in DSSTox (Before)	Total Number of Structures in DSSTox (After)
CASMI 2012 ^1^	14	9	14
CASMI 2013	18 ^2^	8	16
CASMI 2014	42	29	42
CASMI 2016-challenge	208	208	208
CASMI 2016-training	312	312	312
CASMI 2017	243	54	227

^1^ LC-MS data only were considered for the CASMI 2012 contest year. ^2^ 2 additional tautomers for a challenge compound were provided; the total number of unique structures was 16.

**Table 2:** Percentage of the total number of compounds from each CASMI contest year that were ranked in the top 5 by Competitive Fragmentation Modeling for Metabolite Identification (CFM-ID) only and by the summation of CFM-ID and DSSTox Data Source Counts (DS), alongside the percentage in the top 5 reported by the contest years’ winning entry. Complete ranking results are provided in Supplemental File S1.

CASMI Year	CFM-ID Only	CFM-ID + DS	Winners’ Results ^1^	Total in DB/Total in Dataset ^2^
2012	36%	64%	36%	14/14
2013	81%	88%	88%	16/16
2014	57%	76%	71%	42/42
2016-training	63%	96%		312/312
2016-challenge	66%	94%	81%	208/208
2017	59%	53%	74% ^3^	227/243

^1^ When multiple contest categories existed, the winners’ result was taken from the category type scored by allowing participants’ use of all available data (for example, category 3 in the 2016 contest year: http://casmi-contest.org/2016/rules-categories.shtml). ^2^ The number of compounds present in the DSSTox Database (DB) and the number of compounds present in the given CASMI dataset, after dataset assembly review and registration. ^3^ For 2017, in the category allowing only use of in silico fragmentation tools, the winner’s entry got 52% in the top 5.

## Data Availability

CASMI data was accessed via http://casmi-contest.org/ and processed for use in our spectral matching and identification workflow.

## Supplementary Materials

The supplementary materials are available online at https://www.mdpi.com/2218-1989/10/6/260/s1.
