# Supplementary material for: Revisiting Five Years of CASMI Contests with EPA Identification Tools
Source: Metabolites. 2020 Jun 23;10(6):260. doi: 10.3390/metabo10060260 (PMC7345619; doi:10.3390/metabo10060260)
Supplement: Supplementary file 1 [file metabolites-10-00260-s001.zip › Supplemental File 2_v2.docx]

Supplemental File 2

Supplemental Figures for:

*Revisiting Five Years of CASMI Contests with EPA Identification Tools*

McEachran, et al.

Contents: 3 pages, 3 figures


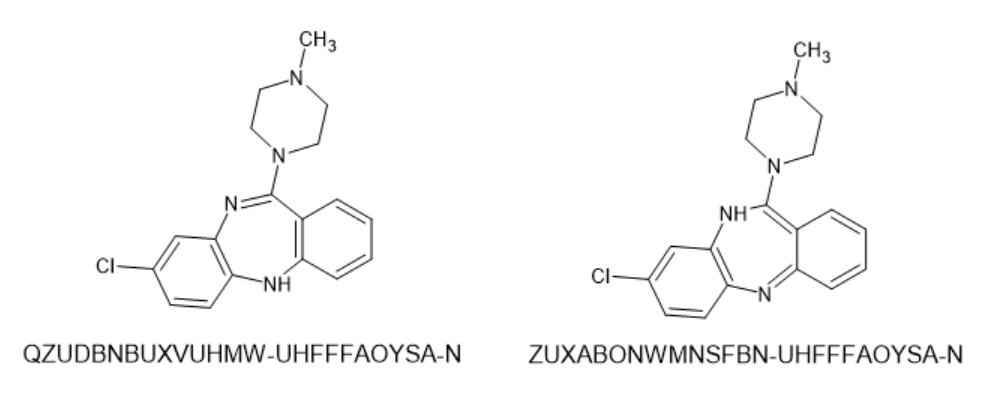


Supplemental Figure S1: Two tautomeric forms of clozapine and the associated standard InChIKeys.


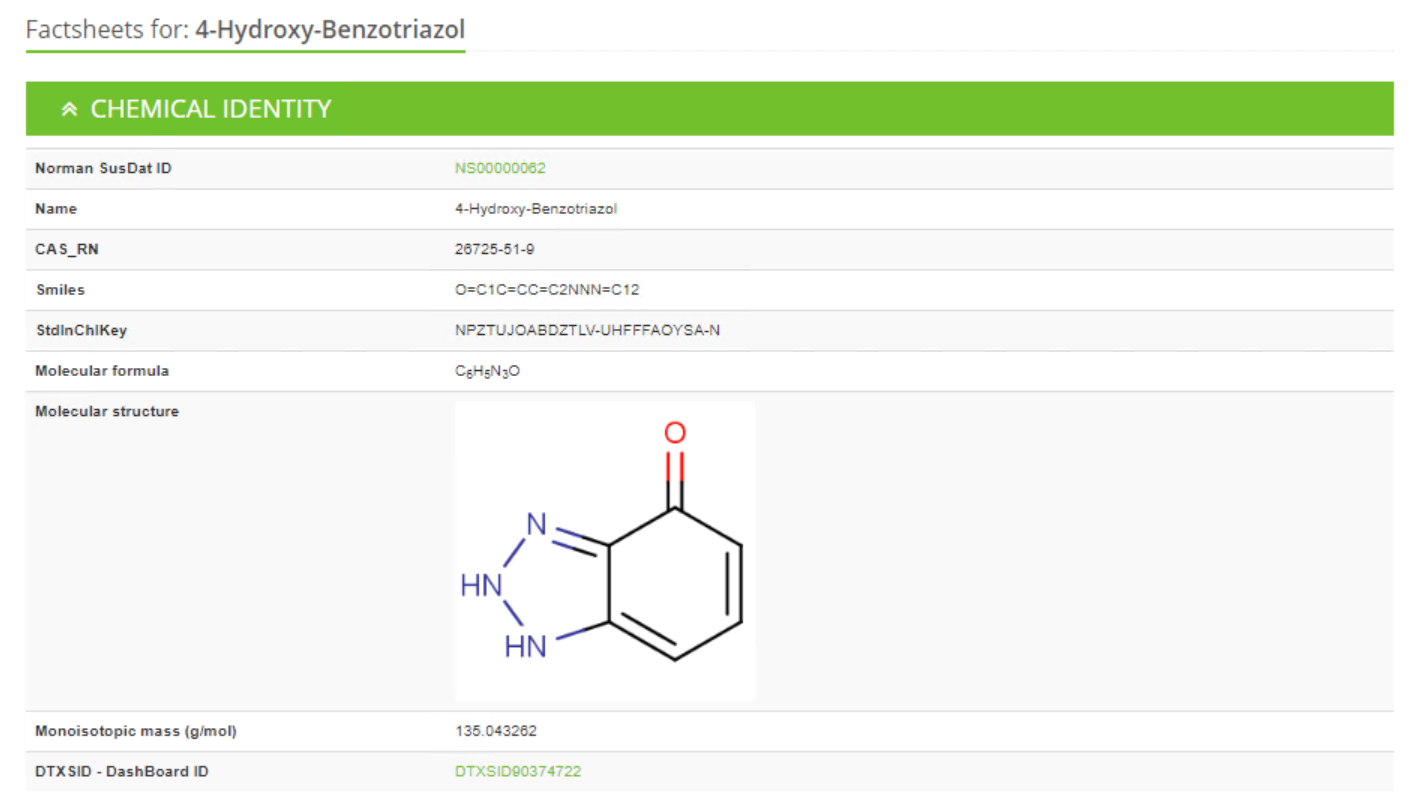


Supplemental Figure S2: The chemical identity and structure representation of 4-hydroxybenzotriazole as shown on the NORMAN Network Factsheets website (<https://www.norman-network.com/nds/factsheets/>). The structural form and associated standard InChIKey is for the keto form of the chemical. The CASRN is for the enol form as represented by <https://comptox.epa.gov/dashboard/dsstoxdb/results?&search=DTXSID90374722>.


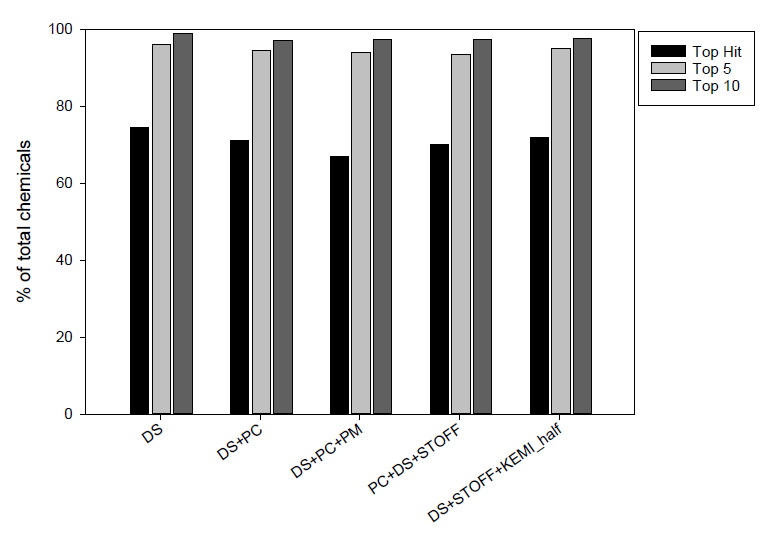


Supplemental Figure S3. Results from a preliminary analysis combining multiple pieces of metadata for identification of 1783 compounds from a variety of sources. Metadata included in the combined rankings are DSSTox Data Sources, PubChem Data Sources, PubMed Reference Counts, presence in the STOFF-IDENT database, and presence in the KEMI list of chemicals expected to exist in the market. Combined metadata rankings do not outperform DS alone.
